# A Review on Dapsone Hypersensitivity Syndrome Among Chinese Patients with an Emphasis on Preventing Adverse Drug Reactions with Genetic Testing

**DOI:** 10.4269/ajtmh.16-0628

**Published:** 2017-05-03

**Authors:** Na Wang, Leela Parimi, Hong Liu, Furen Zhang

**Affiliations:** 1 Department of Dermatology, Shandong Provincial Hospital for Skin Disease, Shandong University, Jinan, China; 2 Department of Dermatology and Venereology, Shandong Provincial Institute of Dermatology and Venereology, Shandong University, Jinan, China; 3 Shandong Provincial Key Laboratory for Dermatovenereology, Jinan, Shandong, China; 4 Shandong Provincial Medical Center for Dermatovenereology, Jinan, Shandong, China

## Abstract

Dapsone is a bactericidal and bacteriostatic against *Mycobacterium leprae*, a causative agent of leprosy. Dapsone is also applied in a range of medical fields because of its anti-inflammatory and immunomodulatory effects. Dapsone hypersensitivity syndrome (DHS) is a rare yet serious adverse drug reaction (ADR) caused by dapsone involving multiple organs. We performed a systematic review of published articles describing dapsone-induced hypersensitivity syndrome, including all Chinese articles and the latest literature available in online databases published between October 2009 and October 2015. We determined the prevalence, clinical characteristics, and mortality rate of DHS. Importantly, we also summarized the recent advances in genetic testing allowing prediction of ADRs. In an initial systematic electronic search, we retrieved 191 articles. Subsequently, these articles were further filtered and ultimately 84 articles (60 Chinese case reports, 21 non-Chinese articles, and three epidemiological studies) were selected, which included 877 patients. The prevalence of DHS among Chinese patients was 1.5% with a fatality rate of 9.6%. Early withdrawal of dapsone and appropriate treatment reduced the fatality rate. Most importantly, genetic screening for the HLA-B*13:01 allele among high-risk populations showed a significant utility as a useful genetic marker to DHS. In conclusion, this review discusses the epidemiological and clinical characteristics of DHS among Chinese patients, which may help physicians to understand this syndrome.

## Introduction

Dapsone (diaminodiphenyl sulfone or DDS) is a sulfone-derived medication that was first used in the clinic as an antibacterial agent to treat leprosy in the 1940s.^1^ In 1981, the World Health Organization began recommending a multidrug therapy (MDT) combination of three drugs: dapsone, rifampicin, and clofazimine, for successful treatment of leprosy. To date, the MDT regimen remains the standard treatment of leprosy. Since the discovery of anti-inflammatory properties of dapsone, it has being used in the treatment of several dermatological and nondermatological inflammatory diseases such as dermatitis herpetiformis, linear IgA bullous dermatosis and chronic bullous dermatosis of childhood, nodulocystic acne, bullous eruption of systemic lupus erythematosus, erythema elevatum diutinum, leukocytoclastic and other kinds of vasculitis, and malaria.^2^–^10^ It is also used as a prophylactic agent against *Pneumocystis pneumonia*, caused by *Pneumocystis jirovecii*, in human immunodeficiency virus patients with CD4 counts below 200/mm^3^.^11^

The adverse effects of dapsone are categorized into two types: 1) dose-dependent (pharmacological) adverse effects that include hemolytic anemia and methemoglobinemia and 2) dose-independent (idiosyncratic) adverse effects that include dapsone hypersensitivity syndrome (DHS). DHS is a severe, multiorgan reaction to dapsone that includes fever, rash, jaundice, lymphadenopathy, splenomegaly, and pedal edema. Hemolytic anemia, atypical lymphocytosis, and hepatitis are other accompanying findings.^6^,^12^ DHS can cause irreversible organ damage or even death if it is not recognized early and managed properly.^9^,^13^

Here, we conducted a systematic review to identify the clinical epidemiology, prognosis, and fatality rate of DHS. Additionally, we also discussed the importance of testing for HLA-B*13:01SNP, which could predict the risk of DHS. Screening for HLA-B*13:01SNP in high-risk populations could greatly reduce DHS incidence and improve the efficacy of dapsone.^14^

## Materials and Methods

### Literature search.

**Figure 1.**
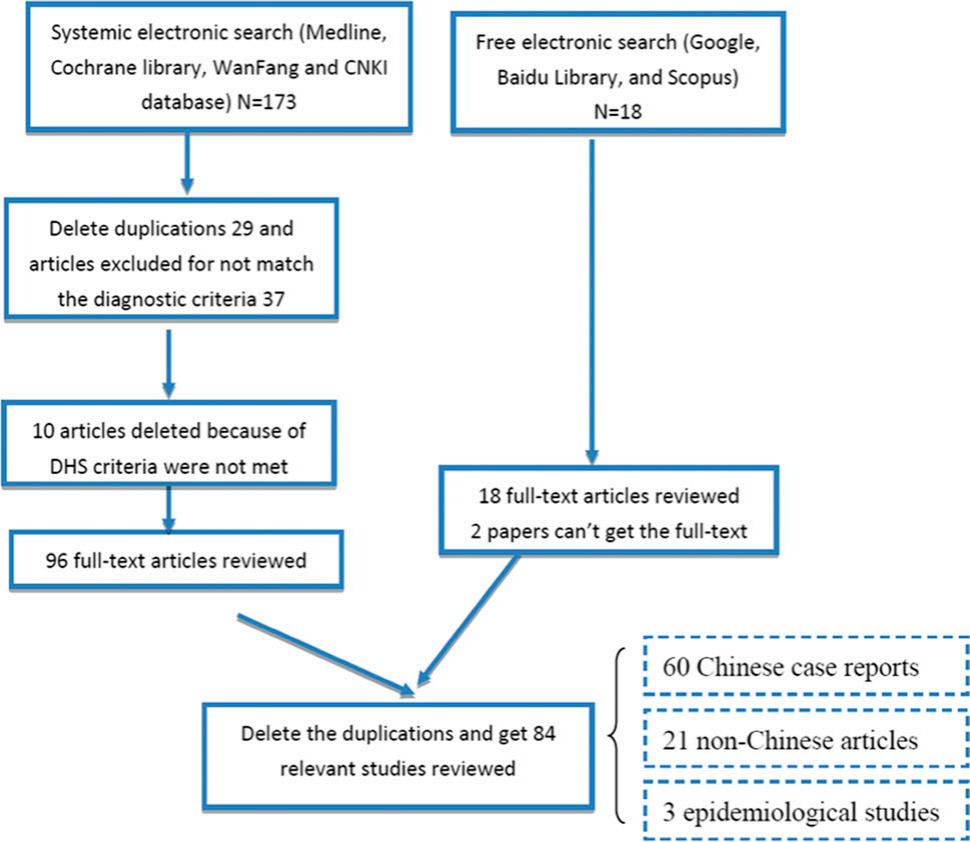
Search terms were used: “dapsone, DHS, dapsone/drug induced hypersensitivity syndrome and Dapsone induced DRESS syndrome.” Identification of relevant studies for inclusion in the systematic review.

A systematic electronic search of all published epidemiological studies and case reports of hypersensitivity reaction to dapsone was conducted using the online databases Medline (via PubMed), Cochrane Library, and Chinese database (such as WanFang 56 data and CNKI 65 databases). The following key words were used: “dapsone, DHS, dapsone/drug induced hypersensitivity syndrome and Dapsone induced DRESS syndrome.” A total of 173 potentially relevant articles were found. In addition, we found 18 relevant articles using a Google search. No publication language restrictions were imposed. A total of 191 articles were found, the majority of which were case reports, including 60 Chinese case reports of 91 patients, and we also retrieved three epidemiological studies, two retrospective studies, and one systematic review. We summarized our search and selection strategy as a flow diagram displayed in Figure 1

.

The following criteria proposed by Richardus and Smith were used for the diagnosis of DHS^2^: 1) presence of at least two of the following signs or symptoms: fever, skin eruption, lymphadenopathy, and liver abnormalities (hepatomegaly, hepatitis, jaundice, and/or deranged liver function tests); 2) appearance of adverse events in the interval between the second and eighth week of dapsone administration, which disappeared upon discontinuation of the drug; 3) symptoms were not attributed to any other simultaneously used drugs or to lepra reactions; and 4) symptoms were not attributed to any other diseases. After excluding the articles that did not meet these four diagnostic criteria, 84 articles remained, including 60 Chinese case reports, 21 non-Chinese articles, and three epidemiological studies (two retrospective studies and one systematic review). In total, these 84 articles included 877 patients; 91 patients included in Chinese case reports, 26 patients included in non-Chinese articles, and 760 patients included in the epidemiological studies.

Our strategy of data extraction from the retrieved articles based on the availability of the following information: study design, patients' characteristics, clinical and preclinical characteristics of DHS, therapy, and outcome (full recovery versus death). Accordingly, based on this data extraction strategy, all subjects included in retrieved articles, with either Chinese or non-Chinese origin, were reviewed and assessed by the first author (Na Wang). Furthermore, random samples of non-Chinese origin were reviewed independently by a second investigator (Leela Parimi), while random samples of Chinese origin, which accounted for more than 15% of whole randomly chosen samples, were assessed by a third investigator (Hong Liu). The agreement between the three reviewers was 99.5%.

To estimate the risk for fatal outcome, Pearson correlation and Fisher's exact test were used to analyze the relationship between sociodemographic factors (sex, age, original disease, and dosage) and prognosis (recovery versus death). All analyses were carried out using SPSS version 17.0 for Windows (SPSS, Chicago, IL).

## Results

Among the Chinese patients, there were 44 males and 47 females. The average age of the patients was 32.3 years, ranging from 8 to 74 years. Half of the cases were diagnosed with leprosy (46/91, 50.5%) (Table 1).

The average latent period of DHS was 30 days, ranging from 3 to 90 days. Complete DHS was reported in 63.7% of patients. In particular, mucosal involvement, pyrexia, skin manifestations (maculopapular rash), hepatic involvement, and lymphadenopathy were observed in 14.3%, 100%, 100%, 93.4%, and 73.6%, respectively (Table 2). Other reported complications were disseminated intravascular coagulation, coagulopathy, and cardiac abnormalities such as complete atrioventricular block (1.1%), myocarditis (2.2%), and pulmonary manifestations such as pneumonia (18.7%). A majority of patients (77/91, 84.6%) were administered 50–100 mg dapsone daily.

In other parts of the world, between October 2009 and October 2015, only 21 case reports, including 26 patients, were reported. As mentioned earlier, we included three epidemiological studies (two retrospective studies and one systematic review). The total number of dapsone users and patients that developed DHS regarding indications, dapsone dosage, and co-medication did not differ significantly between these reports.

The incidence of DHS in the first retrospective study of patients with leprosy treated with MDT between 2006 and 2009 in China was 1.0%.^15^ This study emphasized the need to pay more attention to DHS in patients with leprosy newly treated with the dapsone-containing MDT regimen.

The second retrospective study investigated DHS in patients of different ethnicities and disease indications at the National Taiwan University Hospital between June 2001 and December 2005. The incidence of DHS was 1.66% among patients with nonleprosy. Thus, this study concluded that DHS is less severe in patients with nonleprosy, and hence these patients may have a better prognosis than patients with leprosy.^8^

In addition, Lorenz and others performed a systemic review covering all reported cases of hypersensitivity syndrome to dapsone published between January 1951 and October 2009. Lorenz's review investigated the frequency of DHS, clinical characteristics, risk factors, and fatality rate.^16^ Among 336 patients included in the review, the prevalence of DHS was 1.4% while the fatality rate was 9.9%. Results of the three epidemiological studies are summarized in Table 3. It is important to note that in China dapsone is only available in the form of MDT blister packs and in recent years has, thus, only been prescribed to patients with leprosy. Based on the “National leprosy recording and reporting system,” the total number of leprosy cases in China between 2009 and 2014 were 1,597, 1,324, 1,144, 1,206, 924, and 823, respectively. The incidence of DHS among Chinese patients with leprosy over the last 5 years (2009–2013) was 0.63% (39/6, 195).

Notably, for 52 patients reported between October 2009 and October 2015, we also could collect data on the prevalence and fatality rate of DHS. The prevalence of DHS was 1.5% and the mortality was 9.6%.

As for the 91 Chinese patients, there was no significant association between prognosis of DHS and age (*P* = 0.739), and gender as well (*P* = 0.51). Although, more than half of the patients' have leprosy, there was no significant correlation between leprosy and prognosis of DHS (*P* = 0.315). Also, there was no significant association between dapsone dosage and DHS prognosis (*P* = 0.608).

## Discussion

Drug hypersensitivity, a type of adverse drug reactions (ADRs), remains a major problem for both clinical practice and the pharmaceutical industry. Stevens–Johnson syndrome, toxic epidermal necrolysis, hypersensitivity syndrome, and drug-induced liver injury are examples of idiosyncratic drug reactions associated with significant mortality and morbidity.^16^ These adverse effects have prompted the withdrawal of several newly released drugs from the market, which can be detrimental for the pharmaceutical industry.^17^ DHS is a rare yet potentially life-threatening adverse effect of dapsone associated with a mortality of 9.6% based on reports published in the past 5 years. At present, there are no reliable studies to identify DHS-associated incidence and mortality in China. We first reviewed the published articles and found that the incidence of DHS in the past 5 years in China is 0.63%, which is lower than in previous reports (> 1%).^8^,^18^ This discrepancy might be attributed to the lack of a reliable standard for the diagnosis of DHS and the lack of relevant experience among young Chinese physicians of this rare but severe disease. The 5-year incidence and mortality we calculated was similar to that previously reported by Lorenz and others.^18^

There was no significant association between higher age and fatal outcome of DHS. Frequency of DHS onset may be influenced by the immunological status and genetic markers of patients with leprosy. A review by Lorenz and others highlighted^18^ the link between side effects of leprosy treatment and the high incidence of leprosy in nonaffluent countries. Indeed, we also found that the affluent families are more willing to promptly discontinue dapsone treatment in case of suspected DHS, which reduces the risk of fatal outcome. It worth noting that we found the skin rash severity is associated with the fatal outcome, especially when the skin damage occurred for more than 30% of the body surface area, which also supports previously published reports.^16^ In China, since the last 5 years, the most DHS patients are leprosy; MDT and drugs interaction can also increase fatality; however, rifampicin-related drug hypersensitivity is rare.

On the other hand, in comparison to a study of Taiwanese patients with nonleprosy,^8^ we found a higher rate of mortality and worse prognosis. This discrepancy could be accounted for by the fact that the majority of the patients in the Taiwanese study suffered from noncomplete DHS (358/361, 99.2%), which is a less severe syndrome than complete DHS. Also, Taiwan has a more advanced monitoring network system and more advanced medical care.

A major limitation experienced was the shortage of quality and completeness of presented data in some of included articles, especially Chinese case reports since not all the patients with DHS were reported. Also, some articles in small academic journals are not cited in the CNKI and Wanfang Database, which reduces the accurate estimation of DHS incidence, thus we assessed prevalence instead for the whole patients. Also, for the epidemiological studies, individual patient data were not provided, which made the comparison between all dapsone users and patients with DHS challenging.

Several studies have emphasized the role of HLA in Type B (idiosyncratic) ADRs. HLA, particularly HLA-B, is associated with susceptibility to severe drug hypersensitivity, and together with T-cell receptors plays a major pathogenic role. Ample evidence suggests that HLA is directly involved in drug hypersensitivity.^19^ HLA molecules present antigenic drugs to the T-cell receptor, causing clonal expansion and activation of CD8^+^ cytotoxic T cells.^20^ A pharmacogenomics study found an unusual form of granulysin to be secreted by these cytotoxic T lymphocytes and found natural killer cells to be responsible for the rapid and disseminated keratinocyte death observed in diseases such as Stevens–Johnson syndrome and toxic epidermal necrolysis.^16^

No reliable test capable of predicting the risk for DHS has yet been reported; however, we recently identified a genetic allele, HLA-B*13:01, that is significantly associated with DHS among patients with leprosy.^14^ The HLA-B*13:01 allele sensitively and specifically predicted DHS (85.5% and 85.7%, respectively), and its absence was associated with a 7-fold reduced risk (from 1.4% to 0.2%). This genetic test represents a simple and cost-effective method to determine the risk of developing DHS. In addition, detection of the HLA-B 13:01 risk allele in patients with leprosy using single specific primer-polymerase chain reaction may help physicians to distinguish DHS from lepra reactions that share similar clinical symptoms.

The ethnic-specific genetic association of HLA-B*13:01 with DHS may be due to the difference in allele frequencies among different populations (Table 4). Indeed, ancestry has previously been reported to play an important role in biomarker assessment of drug hypersensitivity.^21^

The association of HLA and ADRs is known to be phenotype specific, so further studies will be required to identify the phenotype specificity of HLA-B*13:01. Clinical screening for biomarkers such as HLA-B 15:02 for carbamazepine and HLA-B 58:01 for allopurinol has significantly reduced ADRs in Taiwan. Therefore, a similar screening program for HLA-B*13:01 needs to be initiated in China to prevent DHS in highly susceptible Chinese patients.

### Implications for future research.

The association of HLA-B 13:01 with DHS once again highlights the role of genetic factors in drug-related adverse reactions. More research needs to done to better characterize such markers, and to develop screening policies that may reduce the morbidity and mortality caused by severe drug reactions. Wider application of pharmacogenomics and translational research in clinical practice may form the basis for development of personalized medicine in the near future.

Further understanding of the mechanisms causing systemic manifestations of DHS, and their relationship with genetic factors, may aid development of new therapeutic strategies in this and other related fields.

## Figures and Tables

**Table 1:** Indications for dapsone usage in Chinese patients

Type of disease	Number of cases	% (Rate)
Leprosy	46	50.5 (46/91)
Bullous disease	7	7.7 (7/91)
Psoriasis	10	11 (10/91)
Purpura	2	2.2 (2/91)
Erythema elevatum diutinum	4	4.4 (4/91)
Other disease	22	24.2 (22/91)
Total	91	100.0

Dapsone can be used to treat several diseases, but currently it is most often used to treat leprosy, which is consistent with the drug prescription in clinics in China. Since the 1990s production of dapsone was stopped in China, only the patients with leprosy have chance to access the free multidrug therapy (MDT, consisting of rifampicin, clofazimine, and dapsone for patients with multibacillary leprosy and rifampicin and dapsone for patients with paucibacillary leprosy) drugs supplied worldwide by the World Health Organization.

**Table 2:** Clinical characteristics of DHS

	Number of patients	%
Systemic		
Pyrexia	91	100
Jaundice	62	68.1
Anemia	57	63.3
Hepatomegaly	44	48.4
Splenomegaly	23	25.3
Pneumonitis	17	18.7
Lymphadenopathy	67	73.6
Liver-enzyme abnormalities	85	93.4
Carditis	5	5.5
Dermatological		
Pruritis	45	49.5
Exfoliative dermatitis	13	14.3
Erythroderma	9	9.9
Maculopapular rash	78	85.7
Mucosal involvement	13	14.3
Management strategy for DHS		
Withdrawal of dapsone	91	100
GCS	79	86.8
GCS plus other drugs	12	13.2

DHS = dapsone hypersensitivity syndrome; GCS = glucocorticoids. Clinical characteristics of DHS for all reviewed patients, including mucosal involvement, pyrexia, skin manifestations, hepatic involvement, and lymphadenopathy, constitute the complete type of DHS. Other complications include cardiac abnormalities, pulmonary manifestations, and anemia.

**Table 3:** Results of three epidemiological studies and our review

Authors	Time interval	Nationality of the study group	Disease category	No. of cases	Mean age (year)	Male:female	Incubation* (day)	Complete DHS ratio†	Incidence of DHS	Death rate (%)
Lorenz and others^16^	September 2009	German	All the patients	336	35.2	1.85	28	Not given	Prevalence 1.4%	9.9
Tian and others^15^	2006–2009	China	Leprosy	63	38	2.15	32.8	13/63 (20.6%)	1.0%	11.1
Sheen and others^8^	July 2001–December 2005	Taiwan	Nonleprosy	361	43.9	0.76	19.5	3/361 (0.83%)	1.66%	0
Wang	October 2009–October 2015	Our review	All the patients	52	34.2	1.26	28.8	26/52 (50%)	Prevalence 1.5%	9.6

DHS = dapsone hypersensitivity syndrome.

* Incubation period is the time between initiation of dapsone administration and occurrence of first hypersensitivity symptoms.

† Complete form of DHS: According to the criteria proposed by Richardus and Smith, there are four diagnostic criteria. Complete DHS is diagnosed in patients presenting all four cardinal symptoms.

**Table 4:** Allele frequencies of HLA-B*13:01 among different ethnic populations

Ethnic population	HLA-B*13:01 (%)
Chinese	2–20
Jiangsu–Zhejiang–Shanghai Han population	> 10
North China	2–5
South China	5–20
Papuans and Australian	28
Indians	1–12
Korean	8.72
Southeast Asians	2–4
Russian	< 1
Northwestern region of Russia	29
Japanese	1.5
Santiago, Chile	< 1
Turkey	18.2
European	0
African	0
